# Genomic Characterization and Phylogenetic Analysis of *HA* and *NA* Genes of Influenza B Virus in Riyadh (2024–2025): Implications for Vaccine Strain Match

**DOI:** 10.3390/v18030358

**Published:** 2026-03-15

**Authors:** Shatha Ata Abdulgader, Ibrahim M. Aziz, Abdulhadi M. Abdulwahed, Reem M. Aljowaie, Mohamed A. Farrag, Abdulaziz M. Almuqrin, Noorah A. Alkubaisi, Fahad N. Almajhdi

**Affiliations:** 1 Department of Clinical Laboratory Sciences, College of Applied Medical Sciences, King Saud University, P.O. Box 10219, Riyadh 12372, Saudi Arabia; 444204318@student.ksu.edu.sa (S.A.A.);; 2 Department of Botany and Microbiology, College of Science, King Saud University, Riyadh 11451, Saudi Arabia; iaziz@ksu.edu.sa (I.M.A.);

**Keywords:** influenza B virus, vaccine effectiveness, N-linked glycosylation, phylogenetic analysis, molecular epidemiology

## Abstract

Background: Influenza B virus (IBV) undergoes continuous genetic mutations that can affect vaccine effectiveness and immune evasion. Although considerable research on IBV epidemiology exists globally, understanding of its genetic behavior in Saudi Arabia remains limited. This study characterized the molecular epidemiology of IBV in Riyadh, Saudi Arabia, during the 2024–2025 influenza season and evaluated compatibility with the current vaccine strain. Methods: Nasopharyngeal samples (*n* = 363) were collected from individuals presenting with influenza-like illness at King Khalid University Hospital in Riyadh. Detection and subtyping of IBV were performed using RT-PCR. Complete sequencing of the *hemagglutinin* (*HA*) and *neuraminidase* (*NA*) genes was conducted on confirmed IBV isolates (*n* = 7), followed by phylogenetic analysis, amino acid substitution mapping, and glycosylation site prediction. Results: Of the 363 samples analyzed, 68 (18.7%) tested positive for IBV, with the majority occurring in adult females aged 15–64 years. Phylogenetic analysis revealed that all seven IBV isolates belonged to the Victoria lineage under subclade V1A.3a.2, corresponding to the current vaccine strain and strains from the 2022–2023 epidemic season. However, molecular analysis identified two substitutions (D129N and D197E) located in antigenic loop-150 and 190-helix, respectively, in the HA polypeptide that distinguished our strains from vaccine strain B/Austria/1359417/2021. Importantly, the N-glycosylation site at position 169 (NKT), which was present in B/Riyadh/1/2010, has been lost in the IBV strains circulating during 2020–2025. Conclusions: While phylogenetic clade compatibility indicates potential vaccine efficacy, the identified amino acid variations and loss of the glycosylation site underscore the necessity for ongoing molecular surveillance to monitor antigenic changes and evaluate vaccine effectiveness within the Saudi Arabian population.

## 1. Introduction

Influenza B virus (IBV) is closely related to influenza A virus (IAV). Consequently, these viruses share similarities in viral structure, genome organization, and epidemiological characteristics. Both are classified within the Orthomyxoviridae family, comprising enveloped, segmented negative-sense RNA viruses with genomes consisting of eight segments: PB2, PB1, PA, HA, NP, NA, MP, and NS [1]. Nevertheless, significant differences exist between them. First, while IAV can infect a wide range of vertebrate hosts, including birds, humans, and various mammals, IBV primarily infects humans. Second, although both virus types cause seasonal respiratory infections in temperate regions and more frequent infections in tropical areas [2], IBV typically has lower prevalence and tends to result in less severe disease compared to IAVs (notably those of the A/H3N2 subtype) [3]. Additionally, while the *HA* gene in A/H3N2 viruses continuously evolves into new antigenic clusters in an episodic fashion [4], IBV is categorized into two main antigenically distinct lineages that circulate concurrently: B/Victoria/2/87-like and B/Yamagata/16/88-like [5]. Notably, since the COVID-19 pandemic, influenza B viruses of the B/Yamagata lineage have not been detected globally in sustained circulation, with no confirmed natural detections reported since March 2020; this apparent absence has led the World Health Organization to conclude that inclusion of a B/Yamagata lineage antigen in seasonal influenza vaccines is no longer warranted, prompting a shift toward trivalent vaccines containing only the B/Victoria lineage component [6].

The B/Victoria lineage was first identified during the 1988/1989 epidemic season, while the B/Yamagata lineage was identified in Japan in May 1988 [7]. In the 1990s, Yamagata-like viruses gained prominence globally, while Victoria-lineage viruses were primarily found in eastern Asia [8]. However, during the 2000/2001 and 2001/2002 influenza seasons, Victoria-lineage viruses reappeared beyond eastern Asia [9]. Since that time, both B lineages have co-circulated during most influenza seasons worldwide [10].

IBV evolution is primarily influenced by genetic drift, which leads to accumulation of mutations that modify antigenic properties and diminish vaccine effectiveness. Numerous studies indicate that these two viral lineages exhibit distinct behaviors within populations, implying that each possesses unique characteristics warranting additional investigation. The main differences observed include patterns of evolutionary escape, variations in prevalence related to temperate or tropical climates, and age-related preferences [11]. Indeed, numerous global studies have identified incompatibility mismatch between circulating IBV strains and current vaccines due to antigenic alterations in key antigenic sites of both IBV lineages [12,13,14,15]. Nonetheless, studies monitoring IBV prevalence and genetic mutations remain limited in Saudi Arabia. The identification of gap mutations (insertions or deletions within viral genome segments) has provided insights into the ongoing viral evolution rather than unique regional adaptation. These structural changes can influence viral fitness, antigenicity, and host immune responses [16].

Studies have investigated the genetic evolution of A/H1N1 and IBV, emphasizing the significant impact of antigenic drift on influenza vaccine efficacy. Investigations into mutations have identified significant amino acid alterations in the HA and NA proteins, especially within antigenic loops that interact with host immune components [17,18]. Furthermore, glycosylation plays a vital role in viral adaptation, influencing receptor interactions, immune detection, and virus survival. Findings suggest that heightened glycosylation in HA can improve immune evasion by concealing antigenic sites, a tactic similarly noted in IBV [19].

From the above studies, IBV plays a major role in the distribution of viral infection. In 2015, IBV, primarily of the Yamagata lineage, constituted 62% of all influenza-related notifications in Australia [20]. A recent study conducted in Saudi Arabia found that, from October 2015 to 2019, the prevalence of IBV was 30.7% over four seasons. The Victoria lineage was dominant during 2015 and 2016, accounting for 64.1%, while the Yamagata lineage became predominant in subsequent seasons (2017 and 2018), with rates of 94.4% and 85.4%, respectively [21]. Our research team previously identified IBV in four out of eighty nasopharyngeal aspirates (NPAs), representing a prevalence rate of 3.8%. Among hospitalized children under five years old in Riyadh, Saudi Arabia, there was notable predominance of the Yamagata lineage, observed in three cases (75%), compared to one case (25%) associated with the Victoria lineage during 2010–2011 [22]. In a more recent study conducted by our group in Riyadh during three epidemic seasons (2020–2023), we reported the presence of IBV in 5.26% of the total 380 samples collected, with predominance of Victoria lineage subclade V1A.3a.2 [16].

Despite these studies conducted in Saudi Arabia, there remains a significant gap in understanding the molecular epidemiology of IBV in the country. The discovery of gap mutations in IBV strains from Saudi Arabia, including deletions in vital antigenic regions, may indicate ongoing evolutionary processes similar to those observed globally [16]. Consequently, this study aimed to explore the genetic diversity and evolutionary trends of IBV in Riyadh over one consecutive season (2024–2025) to elucidate the process of IBV genetic mutations. The research seeks to: (1) identify amino acid substitutions located within critical antigenic regions; (2) assess N-glycosylation profiles; and (3) evaluate the compatibility of vaccine strains with those identified from the Saudi population. Understanding these molecular patterns will provide essential insights for national vaccination strategy, future vaccine strain selection, and preparedness for possible influenza outbreaks in Saudi Arabia.

## 2. Materials and Methods

### 2.1. Ethical Approval

This study was conducted in accordance with the Declaration of Helsinki and approved by the Research Ethics Committee at King Saud University, Riyadh, Saudi Arabia (Institutional Review Board Nos. E-24-9609 and E-25-9609, approved in November 2022 and May 2025, respectively). Informed consent was obtained from all subjects involved in the study.

### 2.2. Clinical Samples

During the influenza season spanning September 2024 to February 2025, a total of 363 nasopharyngeal aspirates were obtained from individuals presenting with symptoms typical of influenza infection, including fatigue, sore throat, headache, cough, runny nose, fever (≥38 °C), and sneezing. These samples were collected from King Khalid University Hospital in Riyadh, Saudi Arabia. Key epidemiological information gathered comprised patient ages, sample collection sites, and dates of sample collection. The aspirates were promptly transported in 2 mL of viral transport medium containing minimal essential medium (MEM) (Gibco, Invitrogen, Grand Island, NY, USA) to the Virology Research Laboratory at the College of Science, King Saud University. Upon arrival, samples were mixed via pulse-vortexing for 15 s, centrifuged at 1000× *g* for 10 min at 4 °C, aliquoted, and stored at −80 °C until further testing.

### 2.3. Detection and Sequencing of IBV Using RT-PCR Assays

The QIAamp Viral RNA Extraction Kit (Qiagen, Hilden, Germany) was utilized for RNA extraction following the manufacturer’s guidelines. IBV was detected using the OneStep Ahead RT-PCR Kit, which includes Taq High-Fidelity DNA Polymerase (Qiagen, Hilden, Germany). The reverse transcription polymerase chain reaction (RT-PCR) was performed using a GeneAmp 9700 thermal cycler (Applied Biosystems, Foster City, CA, USA). The cycling protocol comprised 30 min of reverse transcription at 50 °C, followed by an initial PCR activation step at 95 °C for 15 min. This was succeeded by 40 cycles including denaturation at 94 °C for 30 s, primer annealing at 55 °C for 90 s, and extension at 72 °C for 90 s, concluded with a final extension at 72 °C for 10 min. The resulting PCR products were examined on 1% ethidium bromide-stained agarose gel and compared against a DNA ladder (Qiagen, Hilden, Germany).

Samples testing positive in the detection assays were subjected to subsequent RT-PCR to sequence the complete *HA* and *NA* genes from IBV-positive samples. This procedure employed Taq High-Fidelity DNA Polymerase (Qiagen, Hilden, Germany) along with the primer sets specified in Table 1. Sequencing involved amplifying three overlapping fragments for the entire *HA* gene and two overlapping fragments for the *NA* gene. All amplified segments were sequenced using Sanger sequencing on both strands at Macrogen Inc. (Seoul, Republic of Korea). The Macrogen used standard commercial capillary-based sequencing platforms. Sequence data for both genes were edited and assembled for the selected strains using BioEdit software, version 7.2 (Ibis Biosciences, Carlsbad, CA, USA). The complete sequences were submitted to GenBank and received designated accession numbers PV653673–PV653679 for HA and PV653680–PV653686 for NA.

### 2.4. Phylogenetic and Sequence Data Analysis

A total of 106 IBV strains, emphasizing the *HA* and *NA* genes (Table S1), were obtained from the GenBank and GISAID repositories. These strains represent various viral genotypes, geographical origins, and years of isolation. The consensus sequences for the respective lineages—Victoria and Yamagata—were established as the B/Victoria/02/1987 strain, isolated in Canada in 1987, and the B/Yamagata/16/88 strain, isolated in Japan in 1988. Reference sequences for B/Victoria/02/1987 and the vaccine strain B/Austria/1359417/2021 were obtained from GenBank/GISAID and represent egg-propagated viruses, consistent with standard reference and vaccine prototype material used in influenza surveillance and antigenic characterization. Additionally, the B/Lee/40 strain, initially identified in the United States during the 1940s pandemic, was utilized as a reference point (designated as the “outgroup”) due to its significant co-circulation with both aforementioned strains. Comparisons were conducted between sequences from Riyadh isolates and those of the B/Austria/1359417/2021 vaccine strain as recommended by the World Health Organization (WHO).

**Figure 1:**
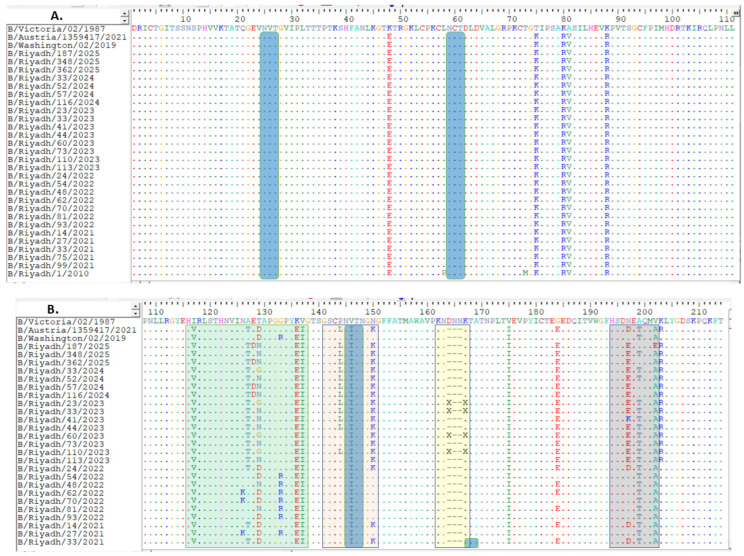

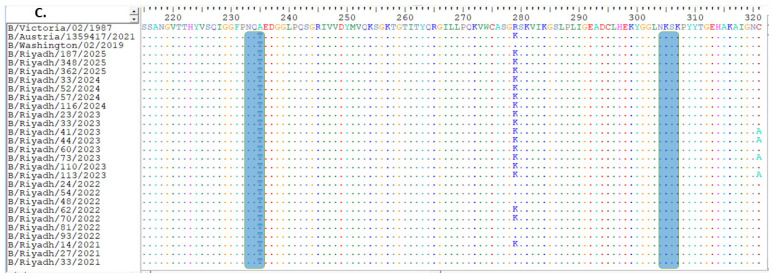
Alignment of deduced amino acid sequences of the *HA* polypeptide: (**A**) site of amino acids from 1 to 111, (**B**) from 112 to 216, (**C**) from 217 to 329. The alignment is conducted utilizing the BioEdit software, version 7.2. Identical residues are marked with dots, while differing residues are represented by their corresponding single-letter codes. The 120-loop (positions 116–137) is highlighted within a green rectangle, the 150-loop (positions 141–150) is indicated by a pink rectangle, the 160-loop (positions 162–167) is enclosed in a yellow rectangle, and the last rectangle at position 190 (positions 194–202) is framed in purple [23]. Predicted N-linked glycosylation sites are denoted by dark blue rectangles. Deletions are indicated relative to the reference strain, with downstream residue numbering maintained.

Multiple sequence alignment for HA and NA polypeptides, along with their respective amino acid sequences and mutation sites, was performed using the ClustalW method through the MegAlign program within Lasergene software version 3.18 (DNAStar Inc., Madison, WI, USA). While amino acid substitutions and glycosylation sites are described throughout the text and figures using mature HA numbering (starting from the first residue of HA1 after signal peptide removal), the sequence alignments presented in Figure 1 retain the full open-reading frame (ORF) numbering to align directly with deposited reference sequences from GenBank (https://www.ncbi.nlm.nih.gov/genbank/, accessed on 12 March 2026) and GISAID (https://gisaid.org/) databases. Positions in the mature protein can be obtained by subtracting the 16-residue signal peptide length from ORF positions. In sequences containing deletions, residue numbering was maintained relative to the reference strain, with deleted positions explicitly indicated, while downstream residue retained their original numbering. No renumbering of residues was performed following deletions. Potential N-linked glycosylation sites (Asn-X-Ser/Thr, where X represents any amino acid except proline) were predicted using NetNGlyc 1.0. Phylogenetic analysis was conducted using the neighbor-joining method in MEGA 11 (v.11, Pennsylvania State University, University Park, PA, USA). A total of 1000 bootstrap replications were performed, and bootstrap values were indicated on the main tree branches.

### 2.5. Statistical Analysis

Statistical analysis was performed using IBM SPSS Statistics version 26.0 (IBM Corp., Armonk, NY, USA). Categorical variables were assessed using Fisher’s exact test. For post hoc analyses, the Z-test with Bonferroni correction was employed. A significance level of *p* < 0.05 was established as the criterion for statistical significance.

## 3. Results

### 3.1. Detection of IBV

The distribution of IBV cases is shown alongside A/H1N1pdm09 and A/H3N2 cases to provide epidemiological context. Among the 363 NPAs collected during the winter season of 2024–2025, 110 (30.3%) were positive for IAV, and 68 (18.7%) were confirmed cases of IBV. Of these 110 positive IAV samples, 68 (61.8%) were identified as A/H1N1pdm09 and 42 (38.2%) as A/H3N2. The number of IBV-positive samples during 2025 was significantly higher, 50 (73.5%), compared to positive cases in 2024 (26.5%). Results also revealed that females had a higher proportion of positive cases (70.5%) compared to males (29.4%). Furthermore, among various age groups, those aged 15–64 years demonstrated a significantly elevated prevalence of IBV (38.2%) compared to other age ranges (Table 2).

### 3.2. Analysis of HA Gene Sequence

The complete sequence of the *HA* gene of isolates obtained from Riyadh during winter season 2024–2025 was 1758 nucleotides. These samples were examined in conjunction with 113 corresponding sequences from global strains obtained from multiple countries. This set included vaccine and reference strains from WHO, accessed through the GenBank and GISAID databases. Upon comparing our strains to the B/Victoria/02/1987 strain, analysis revealed a total of 95-point mutations within the gene. Of these, 20 resulted in amino acid changes, including ten distinct substitutions within the HA1 domain compared with the reference strains B/Victoria/02/1987 and B/Riyadh/1/2010; however, these 12 site substitutions were also observed in strains detected in Riyadh during 2020–2023, as shown in (Figure 1A–C).

Amino acid positions are numbered according to mature influenza B HA1 (after signal peptide cleavage). Approximate correspondences to influenza A/H3 numbering are indicated in brackets for key antigenic regions. Antigenicity of our strains might be influenced due to the location of some mutations in antigenic loops as compare to the B/Victoria/02/1987 strain: I117V, A127T, T129D [120-loop, ≈H3 site E], K136E, and V137I (120-loop), P144L and N150K [150-loop, ≈H3 site A], N197D, A199T, and V202A (190-helix, ≈H3 site B) (Figure 1B) [23].

When analyzing the HA1 domain of our strains with the vaccine strain recommended by WHO for 2024/2025 (B/Austria/1359417/2021), analysis showed the presence of only two amino acid substitutions (D129N [150-loop, ≈H3 site A], and D197E [190-helix, ≈H3 site B]) that were not observed in the current vaccine strain (Figure 1A–C).

### 3.3. Analysis of NA Gene Sequence

**Figure 2:**
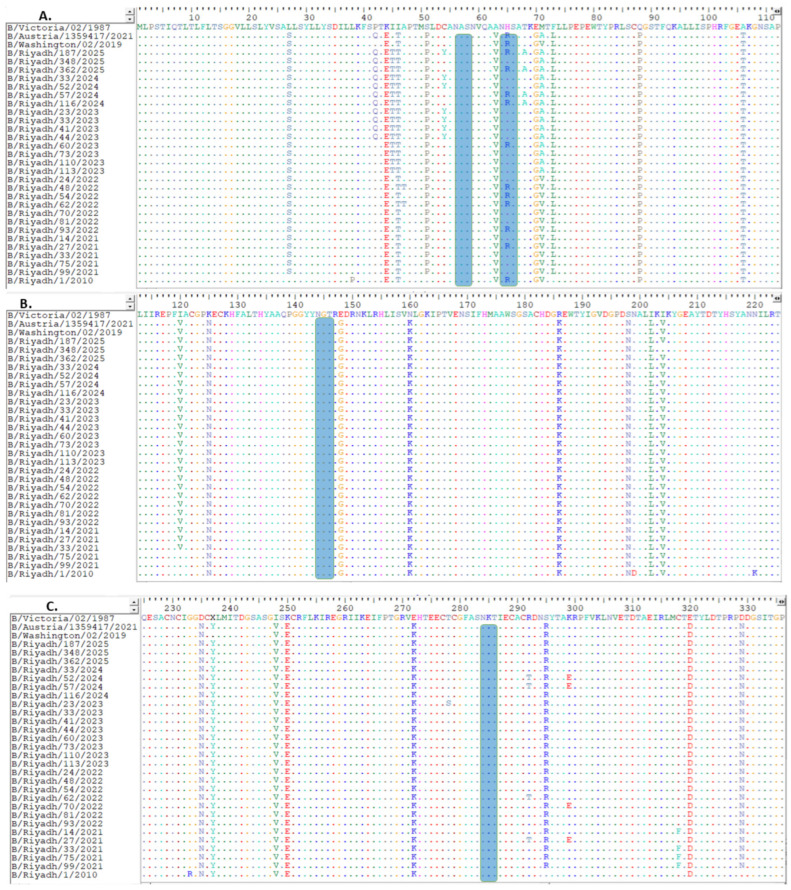

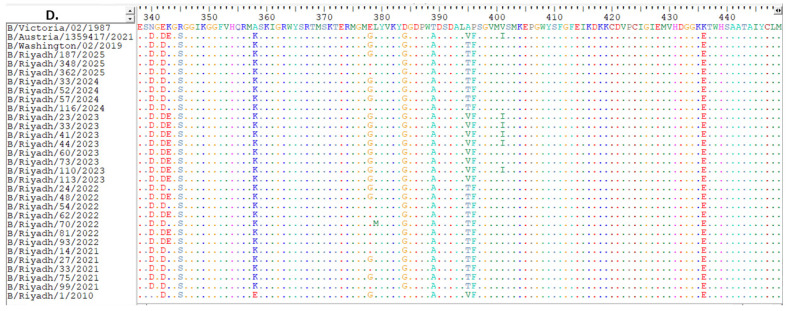
Comparison and alignment of Riyadh IBV strains with vaccine strain B/Austria/1359417/2021: (**A**) location of amino acids from 1 to 112, (**B**) 113 to 224, (**C**) 225 to 336, (**D**) 337 to 449. Changes in amino acids are denoted by upper case letter, while identical amino acids are presented by colored dots. Blue rectangle shows predicted locations of N-glycosylation sites.

The complete length of the *NA* gene sequence of study strains was 1401 nucleotides. These sequences were aligned with corresponding international and vaccine strains. The B/Victoria/02/1987 strain was used as reference to detect amino acid substitutions in our strains. Of a total of 98 mutation sites, 36 were found to cause changes to their corresponding amino acids, and only four were observed to be unique to our strains but absent in both the reference strain and strains identified in previous years. However, some previous strains showed identical mutations in strains from 2021 to 2022 or 2023. Additionally, when compared to the historical strain from 2010 to 2011, analysis revealed the presence of thirteen unique mutations distinguishing our strains from the B/Riyadh/1/2010 isolate (Figure 2A–D).

Comparison of the Riyadh strains with the current vaccine strain (B/Austria/1359417/2021) identified amino acid substitutions at four positions only (I45T, E343K, V395T, and I401V). These substitutions and the corresponding amino acid changes are shown in Figure 2A–D.

### 3.4. Prediction of N-Linked Glycosylation Sites

Regarding N-linked glycosylation sites in the HA1 domain of IBV Riyadh isolates, N-linked glycosylation sites were identified. N-glycosylation sites were limited to only five sites: 25 NVT, 59 NCT, 145 NIT (located within the 150 antigenic loop), and 233 NQT. However, the N-glycosylation site at position 169 (NKT), which was present in B/Riyadh/1/2010, has been lost in the IBV strains circulating during 2020–2025, as well as in the corresponding vaccine strain, as shown in Figure 1A–C.

The NA glycosylation of IBV Riyadh isolates was found to contain only four N-glycosylation sites (56 NAS, 64 NHS, 144 NGT, and 284 NKT).

### 3.5. Phylogenetic Analysis

**Figure 3:**
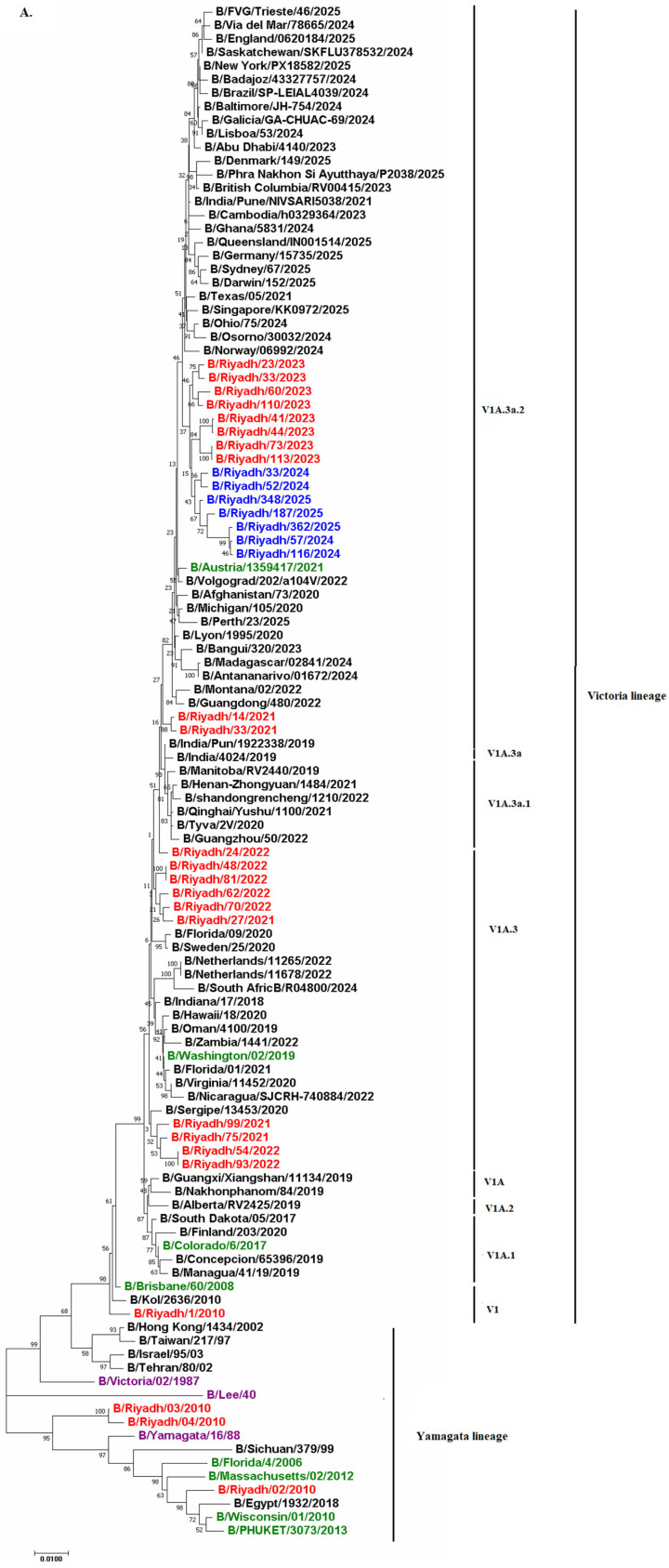

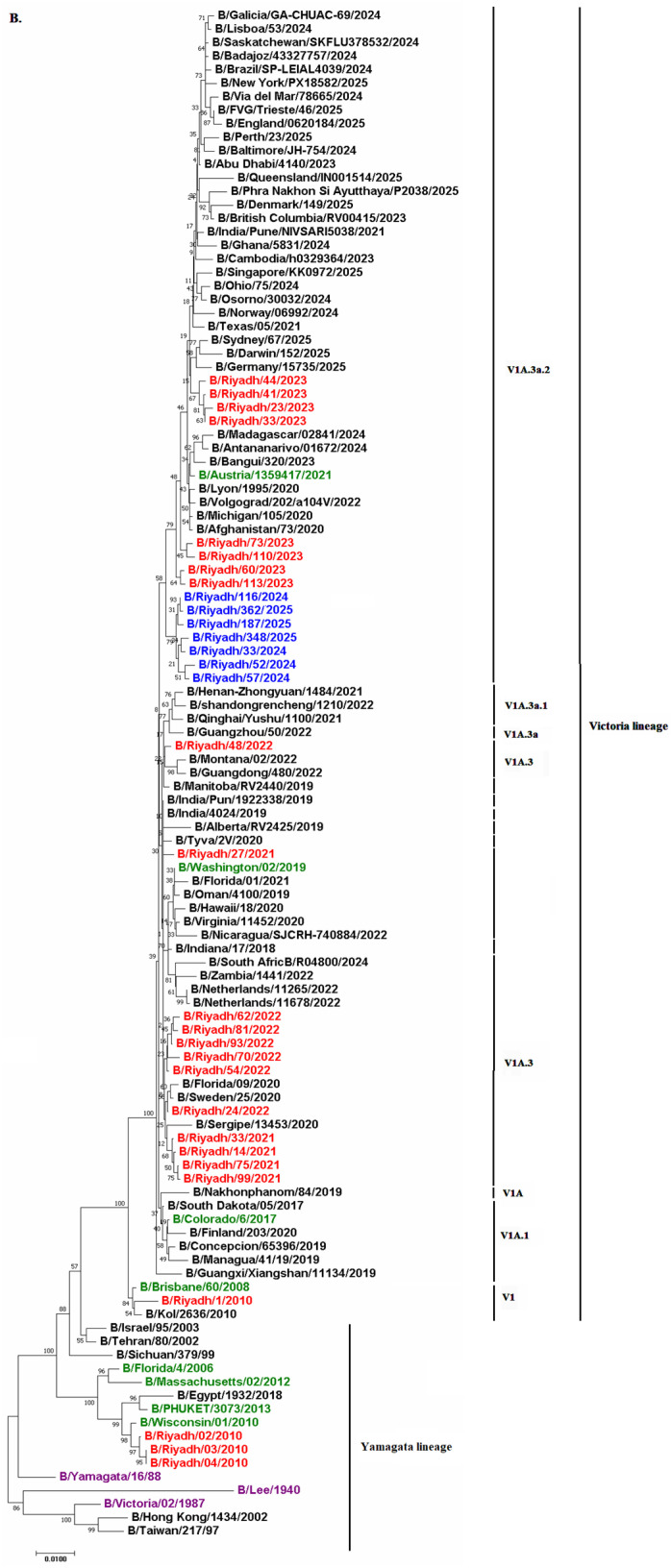
Phylogenetic trees of the (**A**) *HA* and (**B**) *NA* genes of IBV isolates from Riyadh (2024–2025) in comparison with global reference strains, including the vaccine strain B/Austria/1359417/2021. Trees are constructed using the neighbor-joining method with 1000 bootstrap replicates in MEGA 11; bootstrap values are shown on major branches. Riyadh isolates are highlighted in red; the B/Lee/40 outgroup is indicated. Isolates from Riyadh identified in this study are highlighted in blue, while Riyadh isolates identified in previous years are marked in red. The green entries correspond to WHO vaccine strains designated for the Southern Hemisphere. Reference and prototype strains are indicated in purple color.

To determine the lineage of the seven sequences analyzed, phylogenetic analysis of both *HA* and *NA* genes was performed (*n* = 7). Results revealed that all study strains from 2024 to 2025 belonged to the Victoria lineage with sequence homology of 90% to B/Ohio/75/2024 and B/Osorno/30032/2024. Additionally, all study strains circulated within the same clade as the current vaccine strain B/Austria/1359417/2021, which is V1A.3a.2, indicating potential effectiveness of the current vaccine strain against IBV. Comparison of our isolates with previously circulating strains in Riyadh during 2020–2023 indicated that our strains circulated in the same subclade (V1A.3a.2) as Riyadh isolates from 2023. This subclade differed from that of strains circulating during 2021–2022, which showed subclade V1A.3 (Figure 3A,B).

## 4. Discussion

Influenza viruses represent a significant category of respiratory pathogens responsible for serious respiratory infections. Saudi Arabia faces the risk of both imported and emerging viral diseases, attributed to high levels of workforce immigration and the influx of individuals during the annual Hajj pilgrimage from across the globe. This environment may facilitate virus transmission, positioning Saudi Arabia as a potential source of public health concerns and possible outbreaks [22]. Prior to this investigation, our research group reported the circulation of IBV in Riyadh during three consecutive seasons (2020–2023) with strains belonging only to the Victoria lineage; however, they circulated in different subclades, indicating frequent antigenic drift of IBV. Indeed, antigenically drifted strains of IBV may sometimes vary significantly from vaccine strains, resulting in evasion of the immune response generated by annual influenza vaccination [16]. Thus, it is of great importance to monitor IBV circulation patterns in the country during 2024–2025, as no study has reported the prevalence and antigenic patterns of IBV in the country for this period. Accordingly, this study aimed to investigate the epidemiology of IBV in Riyadh during 2024–2025, detect its genetic diversity, and evaluate current influenza vaccine efficacy by comparing circulating strains with the vaccine strain.

In this study, the prevalence of IBV was 18.7%, with most cases being females (70.6%) aged 15–64 years (38.2%). These findings align with a study conducted in Riyadh at King Fahad Medical Research Center during 2019–2020, where IBV was detected in 12% of samples, and most cases were adult females [24]. Similarly, Al-Thaqafi et al. (2021) identified IBV in 30.7% of total samples collected, with most being adult females [21]. However, a study conducted in 2019 at King Fahd Medical Research Center in Jeddah, focusing on pilgrims during the Hajj season, found that 7.4% of patients, predominantly female, were identified with IBV, whereas 92.3% were diagnosed with IAV [25]. Thus, IBV prevalence varies with seasons, with intense viral outbreaks often observed during cold winter months. Additionally, these demographic findings might be associated with workplace exposure and healthcare-seeking patterns rather than inherent vulnerability.

The progression of IBV is marked by persistent genetic alterations, particularly within the HA1 domain of the HA glycoprotein, which is essential for antigenicity and immune recognition [1]. Variations in the HA1 domain, notably in key antigenic loops—specifically the 120-, 150-, 160-, and 190-loops—can lead to considerable antigenic drift, thereby diminishing the effectiveness of immunity established through prior infections or vaccinations [23]. These variations modify crucial epitopes that neutralizing antibodies target, enabling the virus to evade immune detection and resulting in vaccine mismatches [26]. Multiple studies have indicated that amino acid changes in these loops are linked to reduced vaccine effectiveness; for example, Vlaicu et al. [27] found that mutations in the 190-helix (D194E) and the 120-loop antigenic site (E128G and D129N), present in circulating strains compared to vaccine strain B/Austria/1359417/2021, were associated with a decline in vaccine efficacy to 59%. Likewise, another study found that the T214A point mutation located in the 190-helix may influence vaccine effectiveness due to antigenic variation. In certain areas, circulating viruses could differ from WHO-recommended vaccine strains, potentially resulting in reduced protection and inconsistent neutralization responses across different vaccines [28]. Our results revealed 95-point mutations compared to reference strain B/Victoria/02/1987, of which 10 might result in amino acid changes, and only ten mutations were detected in our strains but not found in either the reference strain or strains circulating in Riyadh during 2010–2011. The 12 mutation sites were found in strains identified in Riyadh during 2020–2023 epidemic seasons. Some of these alterations (I117V, A127T, T129D, K136E, and V137I [120-loop, ≈H3 site E], P144L and N150K [150-loop, ≈H3 site A], N197D, A199T, and V202A [190-helix, ≈H3 site B]) were identified in the HA1 antigenic sites, emphasizing the critical need for ongoing surveillance of HA glycoprotein modifications to guide updates in vaccination strategies. Although our findings highlight genetic variations in key regions like the 150-loop, direct antigenic analysis (e.g., using post-infection sera) is essential to confirm any impact on antigenicity. Future studies in Saudi Arabia should prioritize virus isolation and antigenic assays to assess these changes.

In accordance with the studies, we compared our sequences with the current vaccine strain B/Austria/1359417/2021 to assess genetic compatibility and potential antigenic match. Analysis showed four-point mutations, two (D129N [≈H3 site A region], and D197E [≈H3 site B region]) of which were in antigenic loop-150 and 190-helix, respectively. Previous research has shown similar findings in Brazil when comparing strains with the same vaccine strain. Two amino acid changes were detected (D197E and Q200P), found in circulating strains from the 2022–2023 epidemic year [29]. Another study also reported a match between circulating B/Victoria lineage strains and vaccine strain B/Austria/1359417/2021, with only 12 amino acid changes [30]. As such, our results might indicate a match due to the limited number of mutations identified; however, two were in the 150-loop, which could potentially confer resistance to vaccine-induced immunity.

NA inhibitors (NAIs) represent a distinct category of agents that disrupt the sialidase function of the NA glycoprotein. This protein plays a crucial role in cleaving sialic acids from cellular receptors as well as from newly formed HA and NA glycoproteins on emerging virions, thereby enabling release of newly produced viral particles from host cells. Consequently, administration of NAIs—such as oseltamivir, zanamivir, peramivir, and laninamivir—hinders this essential release phase within the viral lifecycle and offers protection to surrounding cells against infection. Currently, these medications are employed as primary treatment options for both influenza A and IBV [31,32,33]. However, amino acid changes within the NA protein can result in drug resistance. This issue has been commonly observed in various regions worldwide and underscores the necessity for vigilant surveillance of evolutionary characteristics that may alter antiviral susceptibility patterns of IAV over time [34,35]. These mutations exhibit wide diversity and can affect various NA protein domains. Not all mutations result in drug resistance; numerous studies have identified different nucleotide and amino acid changes commonly observed in nature that do not significantly contribute to emergence of new drug-resistant strains. Conversely, specific mutations recognized by WHO as frequently associated with resistance to NAIs include R152K, D198E/N/Y, I222L/T, H274Y, N294S, G109E, E105K, and G402S [36]. Additionally, in a study conducted in Australia, researchers identified ten newly emerged mutations in the *NA* of influenza B viruses associated with reduced or highly reduced sensitivity to neuraminidase inhibitors (NAIs), specifically: H101L, G104E, T146P, H439P, H439R, N169S, G247D, and I361V [37]. In this study, ninety-eight mutations were detected compared to reference strain B/Victoria/02/1987, of which thirty-six resulted in amino acid changes, and thirteen were unique to our strains but not found in reference strains. Only four were unique to our strains when compared to strains from 2020 to 2023, although some were observed in strains from 2021 to 2022 or 2023. Two mutations identified in our strains were previously reported to cause resistance to NAIs [38], including N340D and E358K; however, the second mutation in our strains was A358K. Whether this change might render the virus sensitive to NAIs remains unclear and requires further research.

In this study, the number of N-glycosylation sites found in both HA1, and NA glycoproteins were five (25 NVT, 59 NCT, 145 NIT (located within the 150 antigenic loop), and 233 NQT) and four (56 NAS, 64 NHS, 144 NGT, and 284 NKT), respectively, all of which were reported in the vaccine strain and strains identified in previous years (2020–2023 and 2010–2011). Notably, the N-glycosylation site in HA polypeptide at position 169 (NKT) was identified only in B/Riyadh/1/2010 and was lost in strains circulating between 2020 and 2025, despite being conserved in the current vaccine strain B/Austria/1359417/2021, suggesting ongoing genetic divergence-linked glycosylation in HA plays a role in influencing viral infectivity and host immune response. In contrast, NA is affected by glycosylation in terms of its structure, functionality, specificity, virulence, and thermal stability, which subsequently impacts virus release [39]. Glycosylation found on HA and NA proteins helps IAV evade detection by the host immune system by obscuring epitopes responsible for triggering neutralizing antibodies. This can either enhance virus fitness or assist the immune system in recognizing specific strains [40]. Notably, N-linked glycosylation can weaken effectiveness of antibody-mediated cross-neutralization due to antigen masking—a phenomenon evident in both the 1918 influenza pandemic strain and the A(H1N1)pdm09 strain, where additional N-linked glycosylation sites were identified at residues 129 and 163 [41]. Conversely, insufficient N-linked glycosylation of glycoproteins can negatively impact HA protein, resulting in incorrect folding and transport [42]. As a result, it is crucial to monitor these differences in N-linked glycosylation.

Phylogenetic analysis of the *HA* and *NA* genes from strains circulating in Riyadh over multiple seasons (2010–2011, 2020–2023, and 2024–2025) is consistent with the continuous global evolution of influenza B/Victoria lineage viruses, with local strains clustering within internationally circulating subclades rather than forming distinct regional lineages [22]. The phylogenetic clustering of Riyadh isolates within the globally circulating V1A.3a.2 subclade, alongside strains from multiple countries, indicates that the observed substitutions and glycosylation changes are part of the broader worldwide evolution of the B/Victoria lineage rather than a unique local adaptation in Riyadh or Saudi Arabia. Our results showed that all seven IBV isolates clustered within the Victoria lineage with sequence homology of 90% to B/Ohio/75/2024 and B/Osorno/30032/2024. Similar findings were reported in two studies conducted in Riyadh (2020–2023) and Jeddah (2015–2016), showing predominance of the Victoria lineage during the study period [16,21]. However, this result does not align with the study conducted by Ali et al. [22], which reported that the Yamagata lineage was predominant in Riyadh. The shift from B/Yamagata lineage to B/Victoria lineage dominance in Riyadh reflects worldwide patterns, as prevalence of the B/Yamagata lineage has diminished post-2020, likely attributed to reduced transmission during the COVID-19 pandemic [6]. This observation aligns with global surveillance findings indicating that the B/Victoria lineage has been the predominant strain in recent years [43].

Additionally, all study strains circulated within the same clade as current vaccine strain B/Austria/1359417/2021, which is V1A.3a.2, indicating potential effectiveness of the current vaccine strain against IBV. Comparison of our isolates with previously circulating strains in Riyadh during 2020–2023 indicated that our strains circulated in the same subclade (V1A.3a.2) as Riyadh isolates from 2023, while this subclade differed from strains circulating during 2021–2022, which showed subclade V1A.3. The differences in Victoria lineage subclades in the region emphasize the need to monitor IBV in Riyadh to ensure effectiveness of the current vaccine.

The findings indicate that the IBV vaccine component for 2024–2025 was likely phylogenetically related to the circulating strains in Riyadh, which suggests possible match with the vaccine strain. Nevertheless, the detected amino acid changes and loss of N-glycosylation underscore the continuing evolution of the virus and emphasize the importance of ongoing molecular surveillance throughout the year. Notable limitations include a relatively small number of sequenced isolates and the lack of cross-HI testing, which hinders antigenic assessment. Future research should involve larger multicenter sampling, complete genome sequencing using next-generation sequencing to detect mutations and early antiviral resistance, and phenotypic evaluations to more effectively assess vaccine effectiveness and transmission dynamics in the Kingdom. Phylogenetic analysis of *HA* and *NA* genes showed no unique circulation patterns in Saudi Arabia, as our strains clustered with global V1A.3a.2 isolates. While rare local adaptations cannot be ruled out, our data align with worldwide trends in IBV evolution, emphasizing the need for broader regional surveillance to detect any emerging differences.

## 5. Conclusions

This study provides recent molecular insights regarding the prevalence and genetic variation in IBV in Riyadh, Saudi Arabia, during the 2024–2025 influenza season. Of 363 samples examined, IBV constituted 68 cases (18.7%) of all identified influenza virus instances. Phylogenetic analysis revealed that all local isolates belonged to clade V1A.3a.2, corresponding to the WHO-recommended vaccine strain B/Austria/1359417/2021, signifying strong compatibility with the vaccine strain Compared with the reference strain B/Victoria/02/1987, 20 amino acid substitutions were detected in the HA polypeptide, with several localized to HA1 antigenic sites (120-loop, 150-loop, and 190-helix), consistent with ongoing antigenic drift—alongside loss of an N-glycosylation site at position 169 (NKT) of in HA polypeptide in strains circulating between 2020 and 2025—highlight the ongoing molecular evolution of the B/Victoria lineage viruses, as reflected in the strains circulating in Riyadh during the 2024–2025 season.

Although the overall vaccine alignment appears favorable, the detected mutations and loss of glycosylation call for ongoing genomic monitoring and functional assessments to evaluate their impact on antigenicity and vaccine efficacy. Enhancing molecular surveillance initiatives throughout various regions in Saudi Arabia and incorporating serological tests such as hemagglutination inhibition (HI) assays will be crucial. This approach will facilitate early identification of new variants and provide support for evidence-based revisions of seasonal vaccine formulations.

## Figures and Tables

**Table 1:** Primers used in this study.

	Primer Name	Sequence 5′-3′	Product Size (bp)
IBV detection (*NS-2* gene)	IBV-Univ-F	5′-ATG GCC ATC GGA TCC TCA AC-3′	238
	IBV-Univ-R	5′-TGT CAG CTA TTA TGG AGC TG-3′	
Sequencing of *HA* gene	IBV-HA-F1	5′-ATG AAG GCA ATA ATT GTA CTA CTC-3′	440
	IBV-HA-R1	5′-GGC AA G AYC CTG AGG TTC C-3′	
	IBV-HA-F2	5′-CTT TCC TAT AAT GCA CGA CAG AAC-3	677
	IBV-HA-R2	5′-CAT ATT GGG CAA TTT CCT ATG GC-3′	
	IBV-HA-F3	5′-AGC AAG CCT TAC TAC ACA GG-3′	800
	IBV-HA-R3	5′-CCT TAT AGA CAG ATG GAG CAA G-3′	
Sequencing of *NA* gene	IBV-NA-F1	5′-TGA ACA ATG CTA CCT TCA AC-3′	851
	IBV-NA-R1	5′-GCA AAT CCG CAT GAG CAT TC-3′	
	IBV-NA-F2	5′-CAA GAA AGT GCC TGC AAT TGC-3	709
	IBV-NA-R2	5′-GAA CAG AYT CAA CCA TTC CTC C-3′	

**Table 2:** Distribution of samples according to year, gender, and age group.

		No. of Samples N (%)	Positive for IBV	Positive for IAV	Positive for	
			N (%)	N (%)	A/H1N1pdm09 N (%)	A/H3N2 N (%)
Total		363	68 (18.7)	110 (30.3)	68 (61.8)	42 (38.2)
Season	2024	166 (45.7)	18 (26.0)	47 (28.3)	38 (80.8)	9 (19.2)
	2025	197 (54.3)	50 (73.5)	63 (31.9)	30 (47.6)	33 (52.4)
Gender	Male	176 (48.4)	20 (28.9)	45 (25.5)	29 (64.4)	16 (35.5)
	Female	187 (51.5)	48 (70.5)	65 (34.7) ^a^	39 (60) ^a^	26 (39.4)
Age in years	0–4	121 (33.3)	12 (17.6)	27 (22.3)	13 (48.1)	14 (51.8)
	5–14	88 (24.2)	24 (35.2)	29 (32.9)	20 (68.9)	9 (31.0)
	15–64	101 (27.8)	26 (38.2)	33 (32.6) ^b^	16 (48.4)	17 (51.5) ^b^
	≥65	53 (14.6)	6 (8.8)	21 (39.6)	19 (90.4)	2 (9.5)

Note: The data provided are expressed in numerical values (%), ^a^ indicates a significantly greater value (*p* < 0.05) compared to males. ^b^ indicates a significantly greater value (*p* < 0.05) in comparison to the age groups of 0–4, 5–14, and those aged 65 years and older.

## Data Availability

The datasets generated and analyzed during the current study are available from the corresponding author upon reasonable request. The sequence data reported in this study have been deposited in GenBank under accession numbers PV653673–PV653679 for the *HA* gene and PV653680–PV653686 for the *NA* gene.

## Supplementary Materials

The following supporting information can be downloaded at: https://www.mdpi.com/article/10.3390/v18030358/s1, Table S1: Accession numbers of IBV strains included in the sequence and phylogenetic analysis in the current study.
