# Supplementary material for: Genomic Characterization and Phylogenetic Analysis of HA and NA Genes of Influenza B Virus in Riyadh (2024–2025): Implications for Vaccine Strain Match
Source: Viruses. 2026 Mar 15;18(3):358. doi: 10.3390/v18030358 (PMC13030605; doi:10.3390/v18030358)
Supplement: Supplementary file 1 [file viruses-18-00358-s001.zip › viruses-4081582-supplementary.pdf]

Table S1: Accession numbers of IBV strains included in the sequence and phylogenetic analysis in the current study.

No	Strain	Origin	HA	NA	
1.	B/Florida/4/2006	USA	EU515992	CY033878	
2.	B/Brisbane/60/2008	Australia	KX058884	CY073894	
3.	B/Wisconsin/01/2010	USA	KC306166	JN993012	
4.	B/Massachusetts/02/2012	USA	KC891816	KC891815	
5.	B/Phuket/3073/2013	Thailand	EPI1381150	EPI1381149	
6.	B/Colorado/6/2017	USA	EPI1381168	EPI1381167	
7.	B/Lee/40	USA	J02093	J02095	
8.	B/Victoria/02/1987	Canada	CY018757	CY018759	
9.	B/Yamagata/16/88	Japan	M36105	AY139081	
10.	B/Riyadh/01/2010	Saudi Arabia	JN663826	JN663827	
11.	B/Riyadh/02/2010	Saudi Arabia	JQ771075	JQ771076	
12.	B/Riyadh/03/2010	Saudi Arabia	JQ771077	JQ771078	
13.	B/Riyadh/04/2010	Saudi Arabia	JQ771079	KC709817	
14.	B/Hong Kong/1434/2002	Hong Kong	CY018685	CY018687	
15.	B/Israel/95/2003	Israel	AJ784047	AJ784092	
16.	B/Kol/2636/2010	India	JF693249	JF693265	
17.	B/Sichuan/379/99	China	EF566113	AY139080	
18.	B/Tehran/80/2002	Iran	AJ784042	AJ784089	
19.	B/Taiwan/217/97	Taiwan	AY139035	EF584406	
20.	B/Montana/02/2022	USA	OP204851	OP204853	V1A.3a.2
21.	B/Texas/05/2021	USA	OM730213	ON369796	
22.	B/Michigan/105/2020	USA	MW795687	MW795689	
23.	B/Florida/09/2020	USA	MT343013	MW713214	
24.	B/India/Pun/1922338/2019	India	MN945463	MN945448	
25.	B/Indiana/17/2018	USA	MK554872	MK554873	
26.	B/Egypt/1932/2018	Egypt	OL354487	OL354489	

27.	B/South Dakota/05/2017	USA	CY217658	CY217660	
28.	B/Nakhonphanom/84/2019	Thailand	EPI1574052	EPI1574051	V1A
29.	B/Guangxi/Xiangshan/11134/2019	China	EPI1648889	EPI1648888	V1A
30.	B/Managua/41/19/2019	Nicaragua	EPI1421893	EPI1421892	V1A.1
31.	B/Concepcion/65396/2019	Chile	EPI1655914	EPI1655913	V1A.1
32.	B/Alberta/RV2425/2019	Canada	EPI1645174	EPI1645173	V1A.2
33.	B/Washington/02/2019	USA	MK676294	MN155755	V1A.3
34.	B/Hawaii/18/2020	USA	EPI1752206	EPI1752205	V1A.3
35.	B/Oman/4100/2019	Oman	EPI1604127	EPI1604128	V1A.3
36.	B/Sergipe/13453/2020	South America	EPI1806665	EPI1806664	V1A.3
37.	B/Sweden/25/2020	Sweden	EPI1728560	EPI1728559	V1A.3
38.	B/Florida/01/2021	USA	EPI1858734	EPI1858733	V1A.3
39.	B/Zambia/1441/2022	Zambia	EPI2462866	EPI2462859	V1A.3
40.	B/South_AfricB/R04800/2024	South America	EPI3606871	EPI3606869	V1A.3
41.	B/India/4024/2019	India	EPI1664890	EPI1664889	V1A.3a
42.	B/Manitoba/RV2440/2019	Canada	EPI1635729	EPI1635728	V1A.3a
43.	B/Tyva/2V/2020	Russia	EPI1705870	EPI1705872	V1A.3a.1
44.	B/Qinghai/Yushu/1100/2021	China	EPI1878164	EPI1878163	V1A.3a.1
45.	B/shandongrencheng/1210/2022	China	EPI2528159	EPI2528161	V1A.3a.1
46.	B/Henan-Zhongyuan/1484/2021	China	EPI1944637	EPI1944636	V1A.3a.1
47.	B/Austria/1359417/2021	Austria	EPI1926632	EPI1926631	V1A.3a.2
48.	B/Virginia/11452/2020	USA	EPI1711215	EPI1711214	V1A.3
49.	B/Lyon/1995/2020	France	EPI1815360	EPI1815359	V1A.3a.2
50.	B/Afghanistan/73/2020	Afghanistan	EPI1870277	EPI1870276	V1A.3a.2
51.	B/India/Pune/NIVSARI5038/2021	India	EPI2156292	EPI2156291	V1A.3a.2
52.	B/Cambodia/h0329364/2023	Cambodia	EPI2676138	EPI2676137	V1A.3a.2
53.	B/Volgograd/202/a104V/2022	Russia	EPI1981574	EPI1981587	V1A.3a.2

54.	B/Abu_Dhabi/4140/2023	United Arab Emirates	EPI2621569	EPI2621565	V1A.3a.2
55.	B/Bangui/320/2023	Central African Republic	EPI3251993	EPI3251992	V1A.3a.2
56.	B/Badajoz/43327757/2024	Spain	EPI3735511	EPI3735510	V1A.3a.2
57.	B/Ohio/75/2024	North America	EPI3722433	EPI3722432	V1A.3a.2
58.	B/FVG/Trieste/46/2025	Italy	EPI3726377	EPI3726376	V1A.3a.2
**59.**	B/ Singapore/KK0972/2025	Singapore	EPI4546200	EPI4546199	V1A.3a.2
**60.**	B/Brazil/SP-LEIAL4039/2024	Brazil	EPI3810533	EPI3810532	V1A.3a.2
**61.**	B/Perth/23/2025	Australia	EPI4607654	EPI4607649	V1A.3a.2
**62.**	B/Madagascar/02841/2024	Madagascar	EPI4216588	EPI4216587	V1A.3a.2
**63.**	B/Sydney/67/2025	Australia	EPI4582820	EPI4582816	V1A.3a.2
**64.**	B/Queensland/IN001514/2025	Australia	EPI4455656	EPI4455654	V1A.3a.2
**65.**	B/Norway/06992/2024	Norway	EPI3610096	EPI3610095	V1A.3a.2
**66.**	B/England/0620184/2025	United Kingdom	EPI4100845	EPI4100844	V1A.3a.2
**67.**	B/Via_del_Mar/78665/2024	Chile	EPI3771260	EPI3771259	V1A.3a.2
**68.**	B/Galicia/GA-CHUAC-69/2024	Spain	EPI3557507	EPI3557505	V1A.3a.2
**69.**	B/Saskatchewan/SKFLU378532/2024	Canada	EPI3392065	EPI3392064	V1A.3a.2
**70.**	B/Ghana/5831/2024	Ghana	EPI3654606	EPI3654605	V1A.3a.2
**71.**	B/Phra_Nakhon_Si_Ayutthaya/P2038/2025	Thailand	EPI4579528	EPI4579527	V1A.3a.2
**72.**	B/Guangdong/480/2022	China	EPI3453100	EPI3453099	V1A.3a.2
**73.**	B/Antananarivo/01672/2024	Madagascar	EPI3535047	EPI3535043	V1A.3a.2

74.	B/Baltimore/JH-754/2024	USA	EPI3322710	EPI3322709	V1A.3a.2
75.	B/Osorno/30032/2024	Chile	EPI3436966	EPI3436965	V1A.3a.2
76.	B/Netherlands/11265/2022	Netherlands	EPI2027936	EPI2027935	V1A.3
77.	B/Guangzhou/50/2022	China	EPI4400139	EPI4400149	V1A.3a
78.	B/Nicaragua/SJCRH-740884/2022	Nicaraguaa	EPI2971985	EPI2971983	V1A.3
79.	B/Finland/203/2020	Finland	EPI1754217	EPI1754218	V1A.1
80.	B/Netherlands/11678/2022	Netherlands	EPI2285121	EPI2285122	V1A.3
81.	B/Germany/15735/2025	Germany	EPI4162503	EPI4162502	V1A.3a.2
82.	B/Denmark/149/2025	Denmark	EPI4179329	EPI4179326	V1A.3a.2
83.	B/New_York/PX18582/2025	USA	EPI4504813	EPI4504811	
84.	B/British_Columbia/RV00415/2023	Canada	EPI2555718	EPI2555717	V1A.3a.2
85.	B/Lisboa/53/2024	Portugal	EPI3380495	EPI3380494	V1A.3a.2
86.	B/Darwin/152/2025	Australia	EPI4614758	EPI4614754	V1A.3a.2
87.	B/Riyadh/14/2021	Saudi Arabia	PQ895799	PQ895822	
88.	B/Riyadh/27/2021	Saudi Arabia	PQ895800	PQ895823	
89.	B/Riyadh/33/2021	Saudi Arabia	PQ895801	PQ895824	
90.	B/Riyadh/75/2021	Saudi Arabia	PQ895802	PQ895825	
91.	B/Riyadh/99/2021	Saudi Arabia	PQ895803	PQ895826	
92.	B/Riyadh/24/2022	Saudi Arabia	PQ895804	PQ895827	

93.	B/Riyadh/54/2022	Saudi Arabia	PQ895805	PQ895828
94.	B/Riyadh/48/2022	Saudi Arabia	PQ895806	PQ895829
95.	B/Riyadh/62/2022	Saudi Arabia	PQ895807	PQ895830
96.	B/Riyadh/70/2022	Saudi Arabia	PQ895808	PQ895831
97.	B/Riyadh/81/2022	Saudi Arabia	PQ895809	PQ895832
98.	B/Riyadh/93/2022	Saudi Arabia	PQ895810	PQ895833
99.	B/Riyadh/23/2023	Saudi Arabia	PQ895811	PQ895834
100.	B/Riyadh/33/2023	Saudi Arabia	PQ895812	PQ895835
101.	B/Riyadh/41/2023	Saudi Arabia	PQ895813	PQ895836
102.	B/Riyadh/44/2023	Saudi Arabia	PQ895814	PQ895837
103.	B/Riyadh/60/2023	Saudi Arabia	PQ895815	PQ895838
104.	B/Riyadh/73/2023	Saudi Arabia	PQ895816	PQ895839
105.	B/Riyadh/110/2023	Saudi Arabia	PQ895817	Q895840
106.	B/Riyadh/113/2023	Saudi Arabia	PQ895818	Q895841
